# Photocatalytic Activity of Ti-SBA-15/C3N4 for Degradation of 2,4-Dichlorophenoxyacetic Acid in Water under Visible Light

**DOI:** 10.1155/2022/5531219

**Published:** 2022-03-22

**Authors:** Hoa T. T. Duong, Mai T. P. Duong, Oanh K. Nguyen, Son T. Le, Long V. Dang, Binh T. Nguyen, Dang V. Do

**Affiliations:** Faculty of Chemistry, VNU University of Science, Vietnam National University, Ha Noi 19 Le Thanh Tong Hoan Kiem, Hanoi 100000, Vietnam

## Abstract

In the present study, the photocatalytic activity of Ti-SBA-15/C_3_N_4_ catalysts was investigated to degrade 2,4-Dichlorophenoxyacetic acid (2,4-D) herbicides in water under visible light irradiation. The catalysts were synthesized via a simple hydrothermal method and characterized by various analytical techniques, including SAXS, N_2_ adsorption-desorption isotherms, Zeta potential, PL, FT-IR, XRF, TGA, and UV-DRS. Our study indicated that the 2.5Ti-SBA-15/C_3_N_4_ had higher efficiency in the degradation of 2,4-D than Ti-SBA-15 and C_3_N_4_. The decomposition of 2,4-D reached 60% under 180 minutes of visible light irradiation at room temperature on 2.5Ti-SBA-15/C_3_N_4_. Moreover, the degradation of 2,4-D on Ti-SBA-15/C_3_N_4_ was pseudo-first-order kinetics with the highest rate constant (0.00484 min^−1^), which was much higher than that obtained for other photocatalysts reported recently. Furthermore, the catalyst can be reused at least two times for photodegradation of 2,4-D solution under visible light irradiation within a slight decrease in catalytic activity.

## 1. Introduction

Last century, 2,4-Dichlorophenoxyacetic acid (2,4-D) was one of the most generally used herbicides in agricultural producing countries [1, 2]. 2,4-D has primarily been used in agriculture to control weeds in corn and grain, forestry, and lawn care practices [3]. Still, its harmful effects are severe, such as harsh contaminants of superficial and ground waters due to its slow degradation by biological processes, affecting amphibious life in water bodies, causing chromosomal aberrations in human lymphocytes [4, 5]. Therefore, the development of methods to remove wastewater contaminants has gained considerable interest in commercial and academic worldwide. However, most current techniques for the degradation of 2,4-D, such as photo Fenton oxidation and microbial degradation, have to be carried out in a complicated process, producing hazardous by-products or expensive costs [6–8].

Recently, photocatalysis has been a promising advanced water treatment process to replace or support current methods [9]. Titanium-based catalyst has been considered one of the most popular photocatalysts to degrade persistent organic pollutants such as 2,4-D and lindane. Among those, Ti-SBA-15 has been widely used as a catalyst, photocatalyst, and catalyst support for other materials because of its highly ordered and uniform mesoporous channels, thick silica walls, and large surface area [10]. However, several studies indicated that Ti-SBA-15 had limited practical application under visible light conditions because it only absorbed UV light [11, 12]. On the other hand, graphitic carbon nitride (g-C_3_N_4_), a metal-free material with a low electronic bandgap falling in the visible light range of the solar spectrum, has some advantages in photocatalytic applications such as chemically stability under ambient conditions, flexible optical, and non-toxic [13, 14]. g-C_3_N_4_ was also modified by various semiconductors (e g., Ag_2_S, CdS, B_4_O_5_I_2_, Ag_3_PO_4_, Ni_3_C@Ni, ZnO, Bi_2_O_3_–V_2_O_5_) or element doping (N, P, S) to improve its photocatalytic degradation of dye or hydro evolution [15–19]. To the best of our insight, there are no reports on using Ti-SBA-15 modification with carbon nitride for the photodegradation of herbicides 2,4-D under visible light irradiation.

To develop more visible light-efficient catalysts, it is crucial to design photocatalytic systems that are able to operate effectively [20]. Herein, Ti-SBA-15 loading with carbon nitride was prepared and studied for an efficient photocatalyst for the degradation 2,4-D under visible light at neutral pH. The relationship between absorption amount and the photodegradation of 2,4-D on Ti-SBA-15/C_3_N_4_ was discussed in detail. Furthermore, the 2,4-D degradation processes on Ti-SBA-15/C_3_N_4_ have also exchanged views about the kinetics and mechanism in this study.

## 2. Experimental

### 2.1. Chemicals

Pluronic P123 (molecular weight: 5800), Tetraethylorthosilicate (TEOS, (98%wt.)), hydrochloric acid (37%wt.), Titanium (IV) isopropoxide (97%wt.), melamine (99%wt), dicyandiamide (99%wt.), urea (99%wt.), 2,4-D (purity ≥99.0%), and ethanol (99.5%wt.) were purchased from Sigma-Aldrich. All chemicals were used as received without further purification.

### 2.2. Synthesis of xTi-SBA-15

Ti- SBA-15 materials were synthesized by a simple method following the previous reports published anywhere [21]. In an exemplary process, 4.0 g of pluronic P123 was dissolved in 100 mL Deionized (DI) water and 20 ml hydrochloric acid (37 wt.%) under stirring at 40^o^C. After that, 8.0 g of TEOS was added dropwise and stirred for 2 hours. The required amount of Titanium isopropoxide in 10 mL ethanol was dropped slowly into the solution under vigorous stirring at 40^o^C for 24 hours. After aging at 90^o^C for 48 h under steady-state conditions, the as-synthesized material was filtered, washed with DI water, and dried in an oven at 70^o^C for several hours. The material was then calcined at 550^o^C for 6 h in air and denoted as xTi-SBA-15 (*x* is the % wt Ti = 0; 1.0; 2.5; and 5.0).

### 2.3. Synthesis of xTi-SBA-15/C3N4

A certain amount of melamine, urea, dicyandiamide, and xTi-SBA-15 was ground together. This mixture was added to 2 mL deionized water in one neck round-bottom flask under stirring and heating until 85^o^C for 2 hours. Next, water was removed from the mixture by evaporation. Finally, the solid was heated at 550^o^C for 2 h in the inert atmosphere to give a yellow powder denoted as xTi-SBA-15/C_3_N_4_.

### 2.4. Characterization Methods

The FT-IR spectra were obtained using a Bruker ALPHA FT-IR spectrometer. UV-DRS spectra were measured using a PerkinElmer Lambda 365 spectrometer with a reflectance sphere using pure calcium sulfate (CaSO_4_) powder as a white reflection standard. Small-angle X-ray scattering (SAXS) measurements were carried out using a Kratky-type instrument (SAXSess, Anton Paar, Austria) operated at 40 kV and 50 mA in slit collimation using a two-dimensional CCD detector cooled to - 40^o^C. Standard powder X-ray diffraction (XRD) patterns were collected on a Panalytical X'Pert diffractometer (40 kV, 40 mA) equipped with an Xcelerator detector using automatic divergence slits and CuK*α*1 radiation (*λ* = 0.15406 nm) in a 2*θ* scan range between 10 and 50°. X-ray fluorescence (XRF) data have been collected using a Panalytical Epsilon 1 spectrometer operated with an Ag X-ray source. N_2_ adsorption-desorption measurements were carried out at -196^o^C using an ASAP 2020. The zeta potential was analyzed on the ZS90/Malvern system at neutral pH. The photoluminescence (PL) spectra were measured on the HORIBA Jobin Yvon- Fluoromax-4. Thermal gravimetric analysis (TGA) was measured on the Labsys TG/DSC1600 instrument (TMA Setaram, France) with a heating rate of 10^o^C min^−1^ in air.

### 2.5. Degradation of 2,4-D

In a typical experiment, 10 mg catalysts were suspended in 30 mL of the 2,4-D solution with the concentration of 20 mg/L and kept for 90 minutes in the absence of light under stirring to reach adsorption-desorption equilibrium. Then, the mixture was placed into a photoreactor and irradiated by a 1000 W Xenon lamp (LAX 1000, Oriel) equipped with a 90° deflection reflector system (MS 90) containing a dichroic mirror. The distance between the reactor and the deflection reflector system was 12 cm. Experiments were performed with a UV cut-off filter (*λ* ≥ 420 nm, the light intensity: 130 mW cm^−2^). During the irradiation, 2 mL of the liquid was continuously taken from the mixture reaction at a 30 minutes interval for the 2,4-D degradation analysis by High-Performance Liquid Chromatography (HPLC) with the UV-detector using a Lichrosorb RP 18 column (Merck). In addition, a pseudo-first-order model was applied to the study of the degradation reaction kinetics.

## 3. Results and Discussion

### 3.1. Characterization of xTi-SBA-15 and xTi-SBA-15/C3N4

**Figure 1:**
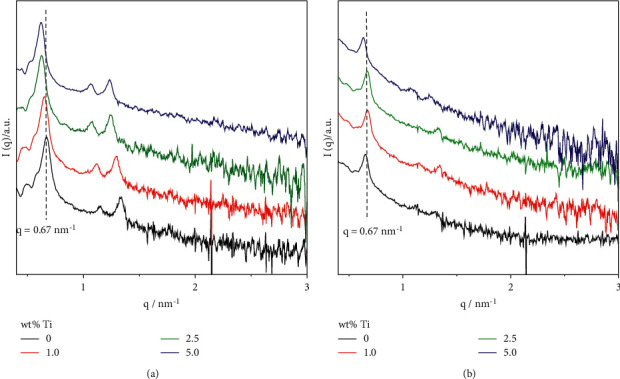
SAXS patterns of (a) xTi-SBA-15 and (b) xTi-SBA-15/C_3_N_4_.

**Figure 2:**
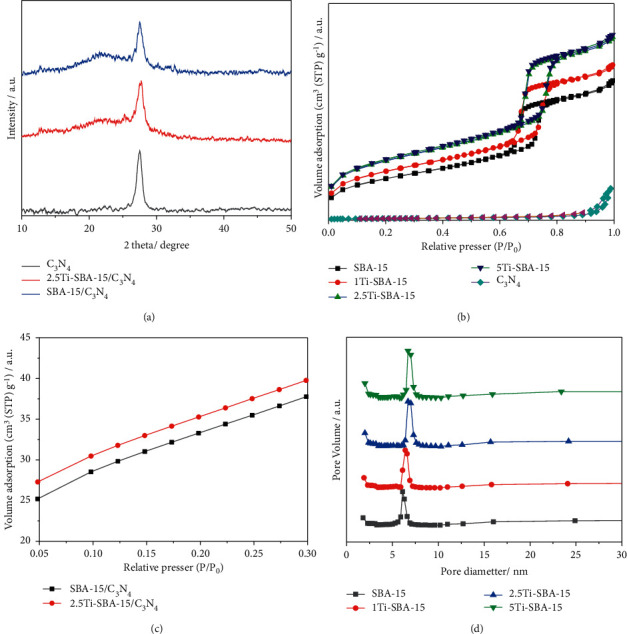
(a) XRD patterns; (b) and (c) Nitrogen sorption isotherms; (d) Pore size distributions of synthesized materials.

Figure 1 shows the SAXS patterns of xTi-SBA-15 and samples modified with C_3_N_4_. Ti-SBA-15 Figure 1(a) has a sharp diffraction peak at *q* = 0.67 nm^−1^ (2 *θ* = 0.94°) due to (100) reflections of a hexagonal mesoporous lattice. In addition, small peaks were also observed at 1 nm^−1^ < *q* < 1.5 nm^−1^ (1.4^o^ < 2 *?* < 2.1^o^), corresponding to (110), (200) reﬂections of the mesopores in this pattern. These results indicate the formation of a hexagonally packed porous structure. For xTi-SBA-15, all features for hexagonal lattice were observed without their slight decrease in peak intensities. The respective q values were also decreased from 0.67 nm^−1^ to 0.621 nm^−1^ along with the increase of the Ti amount. The unit cell parameter was 10.8 nm for pure SBA-15 and increased in the presence of Ti in the porous structure. The difference in the unit cell parameter indicates the larger size of the Ti heteroatom incorporated into SBA-15. It suggested that the long-range order of the mesoporous structure of SBA-15 has remained [22]. Moreover, the SAXS patterns of all xTi-SBA-15/C_3_N_4_ also show hexagonal diffraction peaks due to reflections of (100), (110), and (200) without a slight decrease in intensity.  Figure 1(b), indicating that the structure of these materials has remained after modification. The XRD of xTi-SBA-15/C_3_N_4_ (*x* = 0, 2.5) were also measured at wide-angle and shown in Figure 2(a). The results showed that both materials presented a broad peak at 2*θ* = 27.5^o^, corresponding to the (002) plane of C_3_N_4_ within the decreasing intensity, suggesting that the structure of C_3_N_4_ has also remained after modification by xTi-SBA-15.

The N_2_ adsorption-desorption measurements also confirmed the information on the mesoporous structure Figure 2(b). The typical type IV isotherms were observed with large H1 hysteresis loops at a high relative pressure of 0.6–0.8, corresponding to capillary condensation of nitrogen in the uniform mesopores.  Table 1 and Figure 2(d) show the pore structure character parameters of the synthesized materials. The surface area, pore-volume, and pore size of Ti-SBA-15 increase slightly compared to SBA-15. It could be due to Ti atoms replacing Si atoms and expanding the SBA-15 structure [23, 24]. On the other hand, the surface area decreased quickly after modification due to the presence of C_3_N_4_ within the mesopores Figure 2(c). The amounts of Ti in xTi-SBA-15 (*x* = 1, 2.5, and 5) were 0.9%, 2.5%, and 4.7%, respectively, determined by XRF method.

Electronic and optical properties are of fundamental importance to determine the photoactivity of a catalyst. UV-DRS spectroscopy was applied for characterizing the optical properties of the catalysts.

**Figure 3:**
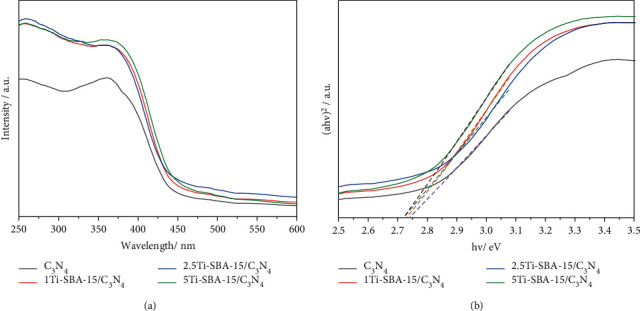
UV-DRS spectra (a) and Tauc plot (b) of pure C_3_N_4_ and xTi-SBA-15/C_3_N_4_.

The weak peak is observed at 256 nm for all the samples xTi-SBA-15/C_3_N_4_ and pure C_3_N_4_, attributed to the aromatic ring's *p*⟶*π∗*Figure 3(a). Besides, the intense peak at 380 nm corresponding to the *n*⟶*π∗* transitions is caused by electron transfer from nonbonding nitrogen orbital to an aromatic anti-bonding orbital. The absorption band of xTi-SBA-15 is slightly toward long-wavelength than C_3_N_4,_ as well as an increase in the absorption of visible light. The Tauc plots Figure 3(b) show the bandgap of xTi-SBA-15 (2.73 eV, 2.72 eV, and 2.72 eV, respectively) slightly decreased in comparison with pure C_3_N_4_ (2.74 eV). The C_3_N_4_ bandgap value corresponds to -1.13 eV, 1.59 eV of the conduction band (CB), and valence band (VB). It is hard to confirm the CB and VB of Ti-SBA-15; however, it is assumed that they were near the TiO_2_ value (-0.5 eV, 2.7 eV of CB and VB, respectively). From these results, it could be demonstrated that the promoted xTi-SBA-15/C_3_N_4_ materials are more active than C_3_N_4_ under visible light irradiation.

**Figure 4:**
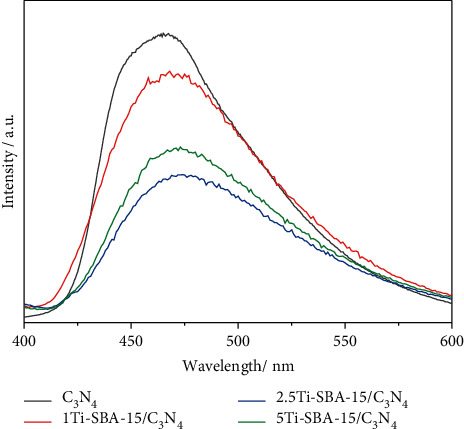
Photoluminescence spectra of pure C_3_N_4_ and xTi-SBA-15/C_3_N_4_.

Photoluminescence (PL) spectra were also measured to analyze photogenerated electron-hole pairs' migration, transfer, and recombination process in the photocatalysts (Figure 4). The emission peak of all materials was at around 470 nm, indicating the structure of C_3_N_4_ has remained after modification. The peak intensity of xTi-SBA-15/C_3_N_4_ decreases along with the increase of Ti amount. It is noticed that the PL intensity of 2.5Ti-SBA-15/C_3_N_4_ was the lowest, corresponding to lower recombination of electron-hole pair.

**Figure 5:**
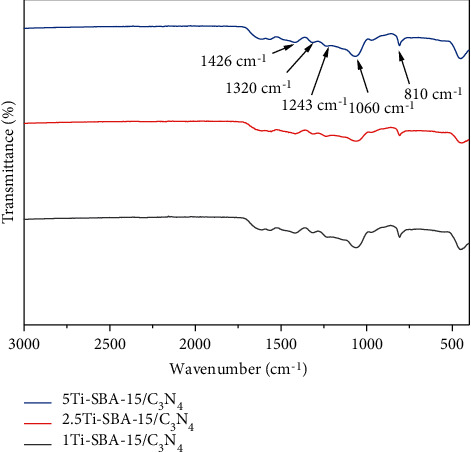
IR spectra of xTi-SBA-15/C_3_N_4_.

The FT-IR spectra of these materials are shown in Figure 5. The results indicated that the carbon nitride replicas show many peaks due to carbon nitride networks' formation by condensing precursors, which can be assigned for C–N heterocycle at 1426 cm^−1^, 1320 cm^−1^, and 1243 cm^−1^ [25]. The band at 1060 cm^−1^ corresponds to Si–O–Si's asymmetric stretching vibration, and the peaks at 810 cm^−1^ can be allocated to the symmetric stretching and distortion forms of Si–O–Si [26]. Additionally, the band at 960 cm^−1^ is generally an identity of Si–O–Ti bonds' existence, as seen in Figure 5.

**Figure 6:**
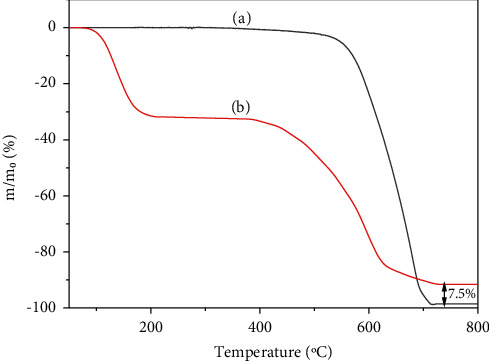
TG analysis of (a) C_3_N_4_; (b) 2.5Ti-SBA-15/C_3_N_4_.

The thermal gravimetric analysis (TGA) was conducted in the air to quantify the relative amount of 2.5Ti-SBA-15 in 2.5Ti-SBA-15/C_3_N_4_ materials. The C_3_N_4_ was still stable at 600^o^C before complete decomposition at 700^o^C. However, the 2.5Ti-SBA-15/C_3_N_4_ lost 30% of weight at 200^o^C, corresponding to the water loss in the pore of 2.5Ti-SBA-15. Then C_3_N_4_ in the composite decomposed entirely at high temperature (>600^o^C), and 7.5% weight corresponds to the remaining 2.5Ti-SBA-15 (Figure 6).

### 3.2. Photodegradation of 2,4-D over Ti-SBA-15/C3N4

The photocatalytic activity of the catalysts was investigated in the photodegradation of 2,4-D in water under visible light irradiation. SBA-15 is known to be inert for 2,4-D degradation under UV light or visible light irradiation, even in the sight of O_2_. By contract, Ti-SBA-15 is reactive and can promote the degradation of 2,4-D in water under UV light irradiation. However, the reaction yield is relatively low, less than 10% compared to C_3_N_4_ (42%) (the data not shown).

**Figure 7:**
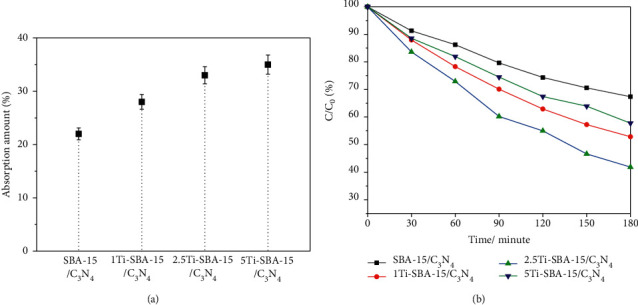
Effects of various amounts of Titanium for 2,4-D removal: (a) light off; (b) light on.

**Figure 8:**
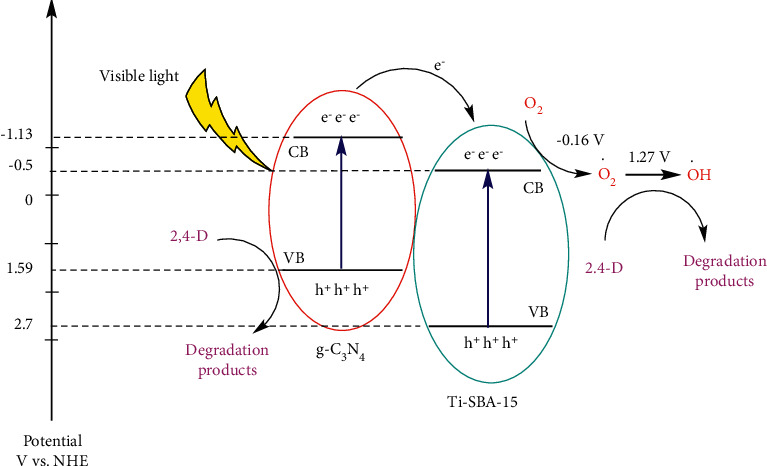
A proposed mechanism diagram of 2,4-D photodegradation on Ti-SBA-15/C_3_N_4_.

The results in Figure 7 demonstrated that all xTi-SBA-15/C_3_N_4_ materials have higher degradation of 2,4-D compared to C_3_N_4_ under visible light irradiation coincident with UV-DRS and PL results. To find a suitable way to explain this phenomenon, the zeta potentials of Ti-SBA-15, SBA-15/C_3_N_4_, and Ti-SBA-15/C_3_N_4_ were -20.7 mV, -20.1 mV, and -13.6 mV, respectively, at pH = 7. The experimental results displayed that the 2,4-D adsorption depends on the amount of Ti in the Ti-SBA-15 Figure 7(a). There is a slight increase in absorption amount from 22% to 35%, together with the raising of Ti amount. It coincides with the result of the N_2_ adsorption-desorption and zeta potential. There is an increase in the photoreaction yield, followed by a significant rise from 0 to 2.5% Ti in materials. The increase in absorption amount also evidenced it. It suggests that the absorption amount might be promotive of the degradation of 2,4-D under visible light irradiation. The 2.5Ti-SBA-15/C_3_N_4_ exhibited the highest photodegradation rate of 2,4-D under visible light up to 60% for 180 minutes of irradiation. In contrast, the highest amount of Ti (in 5Ti-SBA-15/C_3_N_4_) decreased the photodegradation of 2,4-D under visible light irradiation corresponding to UV-DRS and PL data Figure 7(b). There may exist two possible reasons: (i) the Ti-SBA-15 acts as the role of absorber for the 2,4-D, while g-C_3_N_4_ acts as the role of the photocatalyst, (ii) the Ti-SBA-15 acts as the role of absorber and electron migration may exist in the Ti-SBA-15/C_3_N_4_ composite. The photodegradation of SBA-15/C_3_N_4_ was lower than C_3_N_4,_ while the absorption of 2,4-D on a higher surface area material SBA-15/C_3_N_4_ was much higher C_3_N_4_. It was suggested that the first route (i) is not the real reason. The precise cause is still unclear and currently under investigation, and the electron migration exists in the heterojunction of Ti-SBA-15/C_3_N_4_. It was assumed that (i) Ti-SBA-15 absorbed 2,4-D, while C_3_N_4_ absorbed visible light to release electrons in CB (-1.13 eV) and hole (h^+^) in VB; (ii) the electrons transferred to CB of Ti-SBA-15 (−0.5 eV), which is redox O_2_ to O_2_^.^ radical (E(O_2_/O_2_^.^) = − 0.16 V) [27] and OH^.^ radical (E(O_2_^.^/OH^.^) = 1.27 V) [28]. The O_2_^.^, OH^.^ radical or hole (h^+^) of heterojunction of Ti-SBA-15/C_3_N_4_ can oxidize 2,4-D to degradation products like CO_2_ and water, as presented in Figure 8.

**Figure 9:**
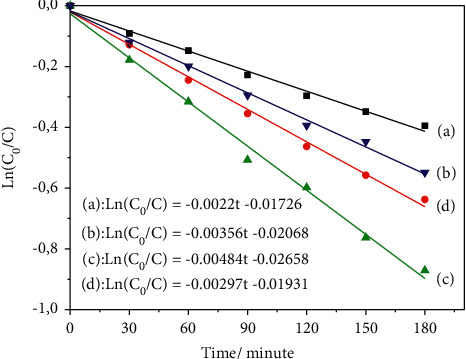
The first-order kinetics of photodegradation of 2,4-D on the catalyst (a): C_3_N_4_; (b): 1Ti-SBA-15/C_3_N_4_; (c): 2.5Ti-SBA-15/C_3_N_4_; (d): 5Ti-SBA-15/C_3_N_4_.

To further study the reaction kinetics of the 2,4-D photodegradation by xTi-SBA-15/C_3_N_4_, the photodegradation data were adapted by implementing a pseudo-first-order model as given by *Ln*(*C*_0_/*C*)=−*kt* , where *k* is the apparent first-order rate constant (min^−1^); C_0_ is the initial concentration at *t* = 0, and C is the concentration at time *t* of the 2,4-D solution.  Figure 9 shows the linear relationship between ln(C_0_/C) and the irradiation time of photodegradation of 2,4-D on the catalysts. It was clearly observed that the photodegradation curve in all cases matched smoothly with pseudo-first-order kinetics. In addition, the apparent rate constants *k* of all the catalysts were estimated and decorated in line with the model (Figure 9). The rate constants *k* values were significantly increased with the rise of Ti loading. The 2.5Ti-SBA-15/C_3_N_4_ catalyst displayed the highest rate constant (0.00484 min^−1^) for 2,4-D degradation, which was much higher than that obtained for other photocatalysts reported recently as ZnO–FeY [29], PdO/Al_2_O_3_–Nd_2_O_3_ [30], chitosan-TiO_2_ beads [31], Fe/TiO_2_ [32] or photo- Fenton process [33].

**Figure 10:**
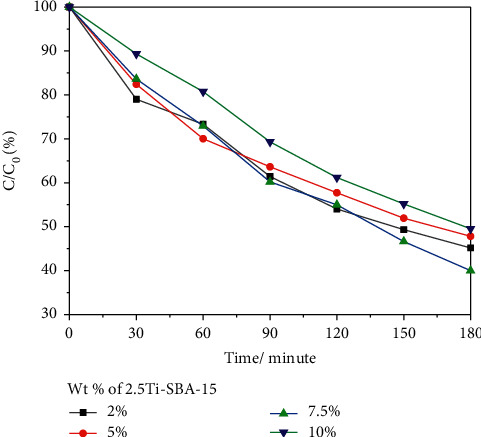
The effect of various amounts of 2.5Ti-SBA-15 in 2.5Ti-SBA-15/C_3_N_4_.

**Figure 11:**
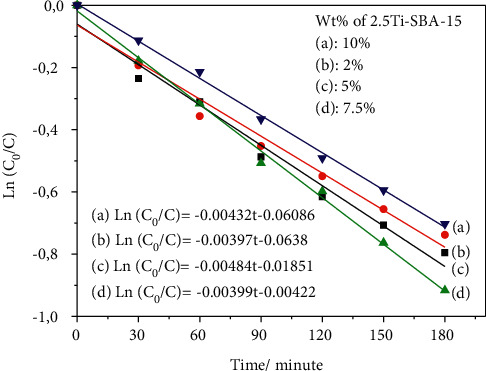
The first-order kinetics of photodegradation of 2,4-D on the catalyst.

To optimize the effect of 2,5Ti-SBA-15 in the composite for 2,4-D photodegradation, numerous composites with different amounts of 2,5Ti-SBA-15 were also prepared.  Figure 10 shows the decomposition of 2,4-D, and Figure 11 displays the relationship between ln(C_0_/C) and the irradiation time of photodegradation depending on the amount of 2,5Ti-SBA-15 in target materials. The photodegradation of 2,4-D under visible light irradiation is increased from 50% (*k* = 0.00397 min^−1^) to 60% (*k* = 0.00484 min^−1^), coincident with the rise of 2,5Ti-SBA-15 from 2% to 7.5%. However, when 2,5Ti-SBA-15 increases up to 10% wt, the photodegradation of 2,4-D slightly decreases, whereas the absorption amount was highest. The decreased absorption of visible light may cause it.

**Figure 12:**
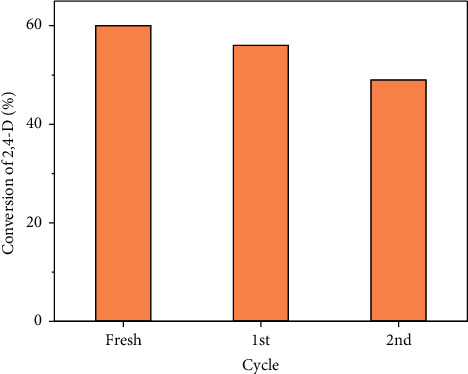
The recycle test of 2,4-D solution photodegradation over 2.5Ti-SBA-15/C_3_N_4_ under visible light irradiation.

Finally, the recycling ability of the 2.5Ti-SBA-15/C_3_N_4_ catalyst was investigated for degradation of the 2,4-D solution under visible light irradiation. The experiments were also carried out using the same procedure without taking the liquid from the mixture reaction. After 180 minutes, the solid was collected by centrifugation and washed with water several times. Then, the solid was dried at 100^o^C and used for the next reaction. The results showed that 2.5Ti-SBA-15/C_3_N_4_ is recycled at least two times with slight losing catalytic activity. The decrease would be ascribed to the loss of catalyst during experiments (Figure 12). From these results, it is demonstrated that the photocatalyst is robust and stable.

## 4. Conclusions

In an effort to develop photocatalysts, this study reported the catalytic activity of Ti-SBA-15/C_3_N_4_ for the degradation of 2,4-D under visible light irradiation. These synthesized materials were characterized by various analytical methods such as SASX, XRD, UV-DRS, N_2_ adsorption-desorption isotherms, Zeta potential, XRF, PL, TGA, and FT-IR. The photodegradation of 2,4-D over Ti-SBA-15/C_3_N_4_ is much higher than pure Ti-SBA-15, SBA-15/C_3_N_4_, or C_3_N_4_. The reaction proceeded to give excellent efficiency on 2.5Ti-SBA-15/C_3_N_4_ with 60% degradation 2,4-D under visible light at room temperature for 180 minutes. The photodegradation of 2,4-D was pseudo-first-order kinetic with the highest constant (*k* = 0.00484 min^−1^), much higher than that obtained for other photocatalysts reported recently. In addition, the catalyst can be reused at least two times within a slight decrease of photocatalytic activities. It was realized that Ti-SBA-15 was an absorber and electron migration exists in the heterojunction of Ti-SBA-15/C_3_N_4_ to enhance the photocatalytic activity of the catalyst. Since a good correlation was established between the adsorption capacity and amount of Ti in Ti-SBA-15/C_3_N_4,_ 2.5Ti-SBA-15 was determined to improve the adsorption property for 90 minutes.

## Figures and Tables

**Table 1:** Pore structure character parameters of the synthesized materials.

Sample	Surface area (m2g-1)	Pore volume (cm3g-1)	Pore size (nm)
SBA-15	695	1.01	5.33
1Ti-SBA-15	811	1.13	5.29
2.5Ti-SBA-15	986	1.35	5.4
5Ti-SBA-15	1011	1.37	5.37
C3N4	26	-	-
SBA-15/C3N4	117	-	-
2.5Ti-SBA-15/C3N4	123	-	-

## Data Availability

The data used to support the findings of this study are included in the article.
